# Monophyly of clade III nematodes is not supported by phylogenetic analysis of complete mitochondrial genome sequences

**DOI:** 10.1186/1471-2164-12-392

**Published:** 2011-08-03

**Authors:** Joong-Ki Park, Tahera Sultana, Sang-Hwa Lee, Seokha Kang, Hyong Kyu Kim, Gi-Sik Min, Keeseon S Eom, Steven A Nadler

**Affiliations:** 1 Graduate Program in Cell Biology and Genetics and Department of Parasitology, College of Medicine, Chungbuk National University, Cheongju 361-763, Korea; 2 Department of Biological Sciences, Inha University, Incheon 402-751, Korea; 3 Graduate Program in Cell Biology and Genetics, College of Medicine, Chungbuk National University, Cheongju 361-763, Korea; 4 Department of Biotechnology and Bioinformatics, College of Science and Technology, Korea University, Chungnam, Korea; 5 Department of Microbiology, College of Medicine, Chungbuk National University, Cheongju 361-763, Korea; 6 Department of Parasitology, College of Medicine, Chungbuk National University, Cheongju 361-763, Korea; 7 Department of Nematology, University of California, Davis, CA 95616, USA

**Keywords:** Mitochondrial genome, Molecular phylogeny, Ascaridida, Spirurida, Oxyurida, Chromadorea, Nematoda

## Abstract

**Background:**

The orders Ascaridida, Oxyurida, and Spirurida represent major components of zooparasitic nematode diversity, including many species of veterinary and medical importance. Phylum-wide nematode phylogenetic hypotheses have mainly been based on nuclear rDNA sequences, but more recently complete mitochondrial (mtDNA) gene sequences have provided another source of molecular information to evaluate relationships. Although there is much agreement between nuclear rDNA and mtDNA phylogenies, relationships among certain major clades are different. In this study we report that mtDNA sequences do not support the monophyly of Ascaridida, Oxyurida and Spirurida (clade III) in contrast to results for nuclear rDNA. Results from mtDNA genomes show promise as an additional independently evolving genome for developing phylogenetic hypotheses for nematodes, although substantially increased taxon sampling is needed for enhanced comparative value with nuclear rDNA. Ultimately, topological incongruence (and congruence) between nuclear rDNA and mtDNA phylogenetic hypotheses will need to be tested relative to additional independent loci that provide appropriate levels of resolution.

**Results:**

For this comparative phylogenetic study, we determined the complete mitochondrial genome sequences of three nematode species, *Cucullanus robustus *(13,972 bp) representing Ascaridida, *Wellcomia siamensis *(14,128 bp) representing Oxyurida, and *Heliconema longissimum *(13,610 bp) representing Spirurida. These new sequences were used along with 33 published nematode mitochondrial genomes to investigate phylogenetic relationships among chromadorean orders. Phylogenetic analyses of both nucleotide and amino acid sequence datasets support the hypothesis that Ascaridida is nested within Rhabditida. The position of Oxyurida within Chromadorea varies among analyses; in most analyses this order is sister to the Ascaridida plus Rhabditida clade, with representative Spirurida forming a distinct clade, however, in one case Oxyurida is sister to Spirurida. Ascaridida, Oxyurida, and Spirurida (the sampled clade III taxa) do not form a monophyletic group based on complete mitochondrial DNA sequences. Tree topology tests revealed that constraining clade III taxa to be monophyletic, given the mtDNA datasets analyzed, was a significantly worse result.

**Conclusion:**

The phylogenetic hypotheses from comparative analysis of the complete mitochondrial genome data (analysis of nucleotide and amino acid datasets, and nucleotide data excluding 3^rd ^positions) indicates that nematodes representing Ascaridida, Oxyurida and Spirurida do not share an exclusive most recent common ancestor, in contrast to published results based on nuclear ribosomal DNA. Overall, mtDNA genome data provides reliable support for nematode relationships that often corroborates findings based on nuclear rDNA. It is anticipated that additional taxonomic sampling will provide a wealth of information on mitochondrial genome evolution and sequence data for developing phylogenetic hypotheses for the phylum Nematoda.

## Background

The phylum Nematoda (roundworms) is an ecologically diverse clade, that has significant impact as parasites of animals and plants, along with providing a diverse array of ecosystem services as free-living species in many environments [1-3]. Parasitic nematodes include many species that negatively impact human health, agricultural production, wildlife, and companion animals [4]. Free-living nematode species occur in almost every environment, particularly soils and aquatic sediments and serve important roles in food webs, particularly as decomposers and predators [5]. Despite their biodiversity, ubiquity, and important impacts, molecular phylogenetic hypotheses for major groups of nematodes, including zooparasitic species, are somewhat lacking when compared to recent advances for other organisms. In addition, prior to the application of molecular data, hypotheses of evolutionary relationships for nematodes relied heavily on interpretation of a limited number of "key morphological characters" or placed excessive emphasis on ecological features; as a result, different investigators recovered different relationships, with resulting debate concerning nematode classification systems (see [6]). During the last 20 years, molecular sequence data have been employed for nematode phylogenetics and have provided testable hypotheses at different levels, from across the phylum to within genera. The vast majority of these studies have been based on gene sequences representing a single nuclear locus, ribosomal DNA (e.g., 18S, 28S, and ITS genes). Such ribosomal DNA studies have included phylum-wide investigations [7-9], studies focused on particular clades identified in phylum-wide investigations (Clade III *sensu *[7];[10]), evaluation of traditional orders or suborders (Cephalobina [11]; Strongylida [12]; Tylenchina [13,14]), and examination of particular genera [15-17]. The first phylum-wide molecular phylogeny of nematodes [7] recognized five major clades. Only one of these groups (clade III) was exclusively composed of animal parasites. This clade represents four traditional orders (Ascaridida, Spirurida, Rhigonematida and Oxyurida), and includes many species of veterinary and medical importance. A revised classification system, based on interpretation of SSU (18S) rDNA phylogenetic trees, has been proposed with each traditional clade III order given infraordinal status within Rhabditida, suborder Spirurina [6,18], and renamed Ascaridomorpha, Spiruromorpha, Rhigonematomorpha and Oxyuridomorpha. Relatively comprehensive sampling and analysis of clade III taxa (103 taxa) using SSU sequences generally supported clade III monophyly (102/103 taxa were monophyletic) and that of Oxyurida, but not monophyly of Ascaridida or Spirurida as traditionally defined [10]. However, relationships among the major clade III orders were not reliably resolved by SSU data, with major differences among phylogenetic trees according to inference method, and inclusion or exclusion of characters judged to be ambiguous with respect to positional (alignment) homology [10].

Analyses of complete mitochondrial genome sequences have provided an alternative hypothesis for relationships among clade III taxa [19-21]. These mitochondrial trees supported a closer relationship between Rhabditida and Ascaridida, with the single oxyurid species in the analysis sister to Rhabditida+Ascaridida, and this latter clade sister to Spirurida. The mtDNA tree topology conflicts with results based on SSU rDNA, which strongly supports monophyly of taxa representing the sampled superfamilies and orders [10]. The recent development of high-throughput approaches for sequencing and annotating mitochondrial genomes [22] holds great promise for increasing the representation of nematode mtDNA genomes for analysis, and has already yielded several new strongylid sequences analyzed herein. However, high-throughput approaches have not yet been applied to increase taxon sampling for clade III nematodes, which remain sparse at most taxonomic levels, including the absence of orders (e.g., clade III, Rhigonematida). The great majority of mitochondrial genome sequences for chromadorean nematodes are derived from the Rhabditida (24 out of 36 species). In contrast, Oxyurida is represented by one species, and although Spirurida and Ascaridida are represented by multiple species, each of these orders includes a single superfamily (Filarioidea and Ascaridoidea). Improving the comparative utility of mtDNA phylogenetics requires broadened taxonomic sampling of nematode mitochondrial genomes.

In this study, we determined the complete mitochondrial genome sequences of three zooparasitic clade III nematode species: *Cucullanus robustus*, the first representative of the superfamily Seuratoidea (Ascaridida), *Wellcomia siamensis*, the second representative of the Oxyurida, and *Heliconema longissimum*, the first representative of the superfamily Physalopteroidea (Spirurida). We also used these data and complete mtDNA sequences from GenBank to infer phylogenetic relationships among major groups of enoplean and chromadorean nematodes.

## Results and discussion

### Gene organization and content

The complete mitochondrial genomes of *C. robustus *(GenBank accession number: GQ332426), *W. siamensis *(GenBank accession number: GQ332427) and *H. longissimum *(GenBank accession number:GQ332423) are 13,972 bp, 14,128 bp and 13,610 bp in length, respectively. The mitochondrial genomes of all three species contain 12 protein coding genes *(cox1-3, nad1-6, nad4L, cob*, and *atp6*), 22 transfer RNA genes and 2 ribosomal RNA genes (*rrnS *and *rrnL*), as found in almost all other nematode mitochondrial genomes reported so far. The exception is *Trichinella spiralis*, which also contains *atp8 *[23]. All 36 genes of each mitochondrial genome are encoded in the same direction with very small intergenic spacer regions (Additional file 1A-C). The genome organizations of each species are shown in Table 1. As previously reported for other nematode mtDNAs, the overall A+T content is very high (Table 2) with average A+T contents of 79.1% for *H. longissimum *(52.9% T, 26.2% A, 6.7% C and 14.1% G), 77.9% for *W. siamensis *(52.6% T, 25.3% A, 4.7% C and 17.4% G), and 71.6% for *C. robustus *(47% T, 24.6% A, 9.1% C and 19.2% G).

**Table 1:** The mitochondrial genome organization of *Cucullanus robustus*(Cr), *Wellcomia siamensis*(Ws) and *Heliconema longissimum*(Hl)

Gene	Cr		Gene	Ws		Gene	Hl	
					
	Positions and lengths of nucleotide sequences (bp)	Initiation and termination codons		Positions and lengths of nucleotide sequences (bp)	Initiation and termination codons		Positions and lengths of nucleotide sequences (bp)	Initiation and termination codons
*cox1*	1 - 1578 (1578)	TTG/TAG	*cox1*	1 - 1558 (1558)	ATA/T	*cox1*	1 - 1593 (1593)	ATT/TAA
*trnC*	1577 - 1633 (57)		*nad1*	1564 - 2424 (861)	ATT/TAG	*trnW*	1644 - 1703 (60)	
*trnM*	1637 - 1690 (54)		*atp6*	2429 - 3029 (601)	TTG/T	*nad6*	1708 - 2145 (438)	ATA/TAA
*trnD*	1693 - 1748 (56)		*trnL2*	3030 - 3084 (55)		*trnR*	2146 - 2201 (56)	
*trnG*	1750 - 1803 (54)		*trnS1*	3093 - 3145 (53)		*trnQ*	2202 - 2256 (55)	
*cox2*	1804 - 2499 (696)	TTG/TAA	*nad2*	3165 - 3987 (823)	TTG/T	*cob*	2257 - 3343 (1087)	ATT/T
*trnH*	2501 - 2555 (55)		*trnI*	3988 - 4049 (62)		*trnL1*	3344 - 3397 (54)	
*rrnL*	2556 - 3513 (958)		*trnY*	4050 - 4106 (57)		*cox3*	3398 - 4174 (777)	ATT/TAA
*nad3*	3514 - 3846 (333)	TTG/TAA	*trnR*	4108 - 4162 (55)		*trnA*	4243 - 4300 (58)	
*nad5*	3850 - 5433 (1584)	ATT/TAA	*trnQ*	4170 - 4224 (55)		*trnL2*	4301 - 4354 (54)	
*nad6*	5558 - 5992 (435)	ATT/TAA	*trnC*	4228 - 4282 (55)		NCR	4355 - 4631 (277)	
*nad4L*	5995 - 6228 (234)	ATT/TAA	*rrnL*	4283 - 5235 (953)		*trnN*	4632 - 4685 (54)	
*trnW*	6229 - 6284 (56)		*trnM*	5236 - 5297 (62)		*trnV*	4686 - 4738 (53)	
*trnA*	6285 - 6338 (54)		*nad6*	5350 - 5784 (435)	TTG/TAG	*trnK*	4739 - 4796 (58)	
*trnE*	6340 - 6393 (54)		*trnV*	5793 - 5846 (54)		*nad4L*	4797 - 5030 (234)	ATA/TAA
*rrnS*	6394 - 7070 (677)		*trnW*	5851 - 5909(59)		*rrnS*	5031 - 5712 (682)	
*trnS2*	7071 - 7122 (52)		*trnF*	5912 - 5967 (56)		*trnY*	5713 - 5768 (56)	
NCR	7123 - 7780 (658)		*cob*	5970 - 7043 (1074)	TTG/TAA	*nad1*	5769 - 6636 (868)	TTG/T
*trnY*	7781 - 7834 (54)		*cox3*	7098 - 7859 (762)	TTG/TAG	*trnF*	6637 - 6693 (57)	
*nad1*	7835 - 8707 (873)	TTG/TAG	*tnrP*	7840 - 7897 (58)		*atp6*	6694 - 7272 (579)	ATT/TAG
*atp6*	8712 - 9311 (600)	ATT/TAA	*trnT*	7901 - 7963 (63)		*trnI*	7273 - 7325 (53)	
*trnK*	9312 - 9372 (61)		*nad4*	7969 - 9192 (1224)	ATT/TAG	*trnG*	7326 - 7384 (59)	
*trnL2*	9375 - 9429 (55)		*trnG*	9195 - 9252 (58)		*cox2*	7385 - 8080 (696)	ATT/TAA
*trnS1*	9430 - 9482 (53)		*nan4L*	9253 - 9486 (234)	TTG/TAG	*trnH*	8083 - 8140 (58)	
*nad2*	9483 - 10325 (843)	TTG/TAA	*trnK*	9508 - 9570 (63)		*rrnL*	8141 - 9113 (973)	
*trnI*	10326 - 10384 (59)		*nad3*	9571 - 9904 (334)	TTG/T	*nad3*	9114 -9440 (327)	ATA/TAG
*trnR*	10387 - 10441 (55)		*nad5*	9905 - 11488 (1584)	TTG/TAG	*trnC*	9441 - 9495 (55)	
*trnQ*	10442 - 10496 (55)		*trnL1*	11494 - 11548 (55)		*trnS2*	9496 - 9547 (52)	
*trnF*	10498 - 10553 (56)		*trnE*	11572 - 11632 (61)		*trnP*	9548 - 9605 (58)	
*cob*	10554 - 11652 (1099)	ATT/T	*trnD*	11648 - 11703 (56)		*trnD*	9609 - 9665 (57)	
*trnL1*	11653 - 11708 (56)		*cox2*	11707 - 12457 (751)	ATT/T	*trnM*	9666 - 9719 (54)	
*cox3*	11709 - 12474 (766)	TTG/T	*trnH*	12498 - 12550 (53)		*nad5*	9725 - 11314 (1590)	ATT/TAG
*trnT*	12475 - 12529 (55)		*rrnS*	12551 - 13282 (732)		*trnE*	11315 - 11372 (58)	
*nad4*	12530 - 13759 (1230)	TTG/TAA	*trnA*	13283 - 13345 (63)		*trnS1*	11403 - 11454 (52)	
*trnV*	13762 - 13815 (54)		*trnS2*	13490 - 13544 (55)		*nad2*	11470 - 12318 (849)	TTG/TAA
*trnN*	13860 - 13916 (57)		NCR	13545 - 14055 (511)		*trnT*	12321 - 12374 (54)	
*trnP*	13917 - 13972 (56)		*trnN*	14056 - 14112 (57)		*nad4*	12377 - 13603 (1227)	ATA/TAA

**Table 2:** Nucleotide composition of *Cucullanus robustus *(Cr), *Wellcomia siamensis *(Ws), *Heliconema longissimum *(Hl) mtDNAs

Nucleotide	Length (bp)			A (%)			C (%)			T (%)			G (%)			A+T (%)		
	
	Cr	Ws	Hl	Cr	Ws	Hl	Cr	Ws	Hl	Cr	Ws	Hl	Cr	Ws	Hl	Cr	Ws	Hl
Entire sequence	13972	14128	13610	24.6	25.3	26.2	9.1	4.7	6.7	47.0	52.6	52.9	19.2	17.4	14.1	71.6	77.9	79.1
Protein coding sequence	10239	10215	10233	20.6	21.4	23.7	9.8	5.0	7.4	49.0	55.5	54.1	20.6	18.1	14.8	69.6	76.9	77.8
Codon position																		
1st	3413	3405	3411	26.7	25.6	27.6	9.2	5.8	7.8	41.9	46.6	46.6	22.2	21.9	17.9	68.6	72.3	74.2
2nd	3143	3405	3411	17.5	17.7	19.0	13.2	8.6	11.9	52.6	55.9	51.9	16.6	17.7	17.2	70.1	73.6	70.9
3rd	3413	3405	3411	17.7	20.9	24.4	6.9	0.6	2.5	52.6	63.8	63.8	22.8	14.7	9.4	70.3	84.7	88.2
Ribosomal RNA gene sequence	1635	1685	1655	33.9	33.5	31.3	7.1	4.3	5.4	42.9	45.8	50.0	16.1	16.4	13.3	76.8	79.3	81.3
Transfer RNA gene sequence	1217	1265	1223	34.3	35.1	33.9	8.1	4.3	4.9	38.9	44.6	48.3	18.7	16.0	12.8	73.2	79.7	82.2
Non coding region	658	511	277	43.0	48.9	51.6	4.6	2.3	1.8	44.4	43.4	45.1	8.1	5.3	1.4	87.4	92.3	96.7

*Termination codons were not included.

### Protein-coding genes and codon usage

Twelve protein-coding genes were identified for each of the three species (Table 1), ranging in size from 234 bp (*nad4L*) for *C. robustus, W. siamensis *and *H. longissimum *to 1,593 bp (*cox1*) for *H. longissimum*. For the 12 protein-coding genes of *C. robustus*, five (*atp6, cob, nad4L, nad5 *and *nad6*) are inferred to use ATT as the start codon, whereas the other seven (*cox1, cox2, cox3, nad1, nad2, nad3 *and *nad4*) use TTG. In *H. longissimum*, six genes (*cox1, cox2, cox3, nad5, cob *and *atp6*) are inferred to start with ATT, four others (*nad3, nad4, nad4L *and *nad6*) start with ATA, and *nad1 *and *nad2 *use TTG as the start codon. In *W. siamensis*, eight genes (*cox3, nad2, nad3, nad4L, nad5, nad6, atp6 *and *cob*) are inferred to use TTG as the start codon, three genes (*cox2*, *nad1 *and *nad4*) start with ATT, and *cox1 *uses ATA as the start codon. For *C. robustus*, eight genes are inferred to use TAA as the termination codon (*cox2, nad2, nad3, nad4, nad4L, nad5, nad6 *and *atp6*) and two use TAG (*cox1 *and *nad1*). In *H. longissimum*, TAA is also the most commonly used termination codon (*cox1*, *cox2, nad2, cox3, nad4, nad4L *and *nad6*) whereas TAG is used for three genes (*nad3*, *nad5 *and *atp6*). For *W. siamensis*, TAG is used as the termination codon for six genes (*cox3, nad1, nad4, nad4L, nad5 *and *nad6*), whereas TAA is used only for the *cob *gene. Termination with the incomplete codon T is inferred for *cox3 *and *cob *of *C. robustus *and *nad1 *and *cob *of *H. longissimum*. The incomplete termination codon T is used in high frequency (five of 12 genes) for *W. siamensis *(*cox1, cox2, nad2, nad3 *and *atp6*). Start and termination codons are shown for each species in Table 1. The protein-coding genes of the three mitochondrial genomes are composed of amino acids that are encoded by T-rich codons, as has been previously documented for all major clades of chromadorean nematodes (*Onchocerca volvulus*, Spirurida [24]; *Strongyloides stercoralis*, Rhabditida [19]; *Anisakis simplex*, Ascaridida [20]; *Toxocara *spp., Ascaridida [25]; *Enterobius vermicularis*, Oxyurida [21]). The six most frequently used codons are all T-rich (having more than two Ts in a triplet): TTT (13.1%), TTA (6.91%), TTG (6.56%), ATT (5.71%), GTT (5.1%), and TCT (4.48%) in *C. robustus*; TTT (18.88%), TTA (8.78%), ATT (7.55%), GTT (7.11%), TTG (6.08%), and TAT (5.02%) in *W. siamensis*; TTT (18.0%), TTA (9.41%), ATT (8.82%), TAT (6.01%), GTT (3.96%), and TCT (3.69%) in *H. longissimum*. These T-rich codons account for almost half of all codons used (Additional file 2) in these three species (43.3% in *C. robustus*, 53.4% in *W. siamensis*, and 49.9% in *H. longissimum*). In addition, unequal use of synonymous codons, especially bias against codons with C in the third position is very prominent in four-fold and two-fold degenerate codon families. For instance, the relative frequency of phenylalanine encoded by TTC is strikingly decreased in each species (1.26% in *C. robustus*, 0.06% in *W. siamensis*, and 0.35% in *H. longissimum*). These factors (higher frequency of T-rich codons and unequal usage of synonymous codons with bias against C-rich codons) correlate with the high percentage of A+T content in the nucleotide composition of protein-coding genes (A+T content of 69.6%, 76.9%, and 77.8% for *C. robustus, W. siamensis*, and *H. longissimum*, respectively; Table 2).

### Ribosomal RNA and Transfer rRNA genes

The small subunit ribosomal RNA (*rrnS*) and large subunit ribosomal RNA (*rrnL*) of each mtDNA were initially identified by comparison with related species of the same orders. It was not possible to confirm the exact starting and ending positions of the two ribosomal RNA genes, therefore the entire flanking regions between the boundaries of their respective adjoining genes were estimated as *rrnS *and *rrnL *in each species (Table 1). The *rrnS *of *C. robustus, W. siamensis *and *H. longissimum *is 677 bp, 732 bp, and 682 bp in size, respectively. The total length of *rrnL *is 958 bp in *C. robustus*, 953 bp in *W. siamensis*, and 973 bp in *H. longissimum*. The predicted secondary structures of 22 tRNAs for each of the three species (Additional file 3) are similar to those reported from a variety of other nematode species (except *Trichinella spiralis*; [23]). For example, all these tRNAs, except for *trnS1 *and *trnS2*, lack a TΨC and instead are equipped with a TV-replacement loop; in addition, *trnS1 *and *trnS2 *lack a dihydrouridine (DHU) arm, but have a TΨC stem-loop structure [19,20,24,26-29].

### Non-coding region

The AT-rich non-coding regions (NCR) for *C. robustus, W. siamensis *and *H. longissimum *are, respectively, 658 bp (located between *trnS2 *and *trnY*), 511 bp (located between *trnS2 *and *trnN*), and 277 bp (located between *trnL2 *and *trnN*) in length (Table 1). These NCR regions have much higher A+T content than any other regions of these mitochondrial genomes (Table 2), with A+T contents of 87.4% (*C. robustus*), 96.7% (*H. longissimum*) and 92.3% (*W. siamensis*).

### Nematode Mitochondrial phylogeny

**Figure 1:**
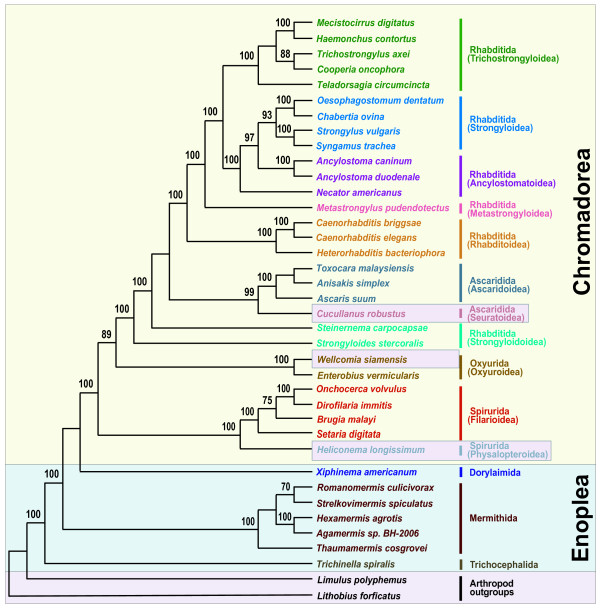
**Single maximum likelihood tree with values from the separate bootstrap analysis shown at internal nodes when 70% or greater**. Analysis of nucleotide sequences for 12 protein-coding genes (11,727 characters) for 36 nematode mitochondrial genomes inferred using RAxML (see methods for analysis details). This single tree of highest likelihood (score ln -267462.174376) is fully resolved, whereas the bootstrap majority-rule consensus tree (not shown) is not.

**Figure 2:**
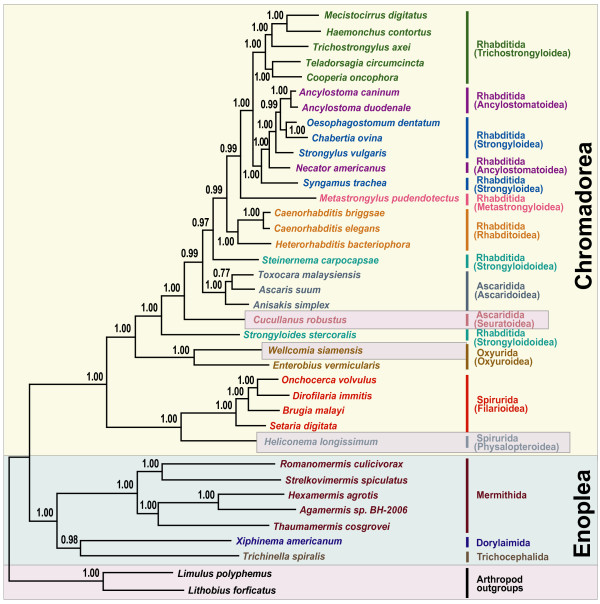
**Phylogenetic tree from Bayesian analysis of amino sequences for 12 protein-coding genes for 36 nematode mitochondrial genomes**. Bayesian posterior probability values (BPP), shown above the nodes, were estimated after the initial 200 trees (the first 2 × 10^5 ^generations) were discarded as burn-in (see methods for analysis details).

**Figure 3:**
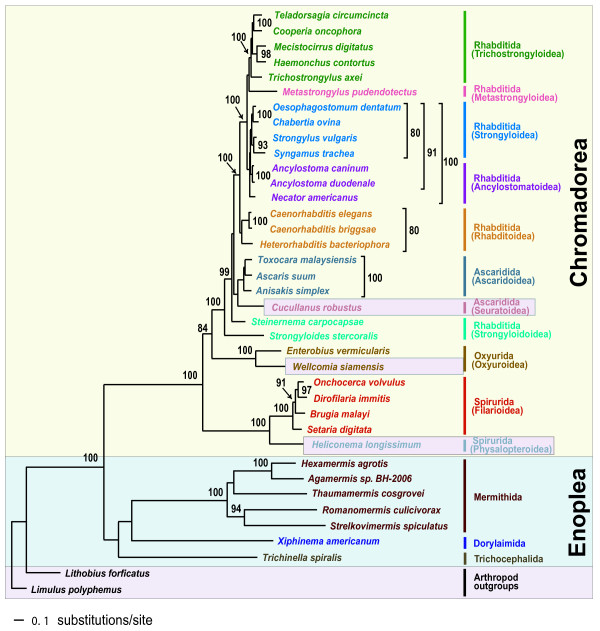
**Single maximum likelihood tree with values from the separate bootstrap analysis shown at internal nodes when 70% or greater**. Analysis of nucleotide sequences for 12 protein-coding genes with third codon positions removed from the dataset (7,818 characters). This single tree of highest likelihood (score ln -141613.521771) is fully resolved (see methods for analysis details).

**Figure 4:**
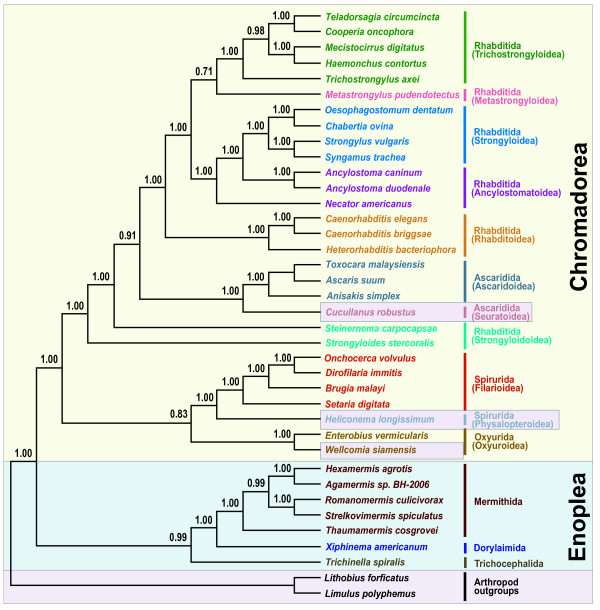
**Phylogenetic tree from Bayesian analysis of nucleotide sequences for 12 protein-coding genes with third codon positions removed (7,818 characters)**. The best-fit substitution model for each of 12 genes was estimated using the AIC criterion implemented in MrModeltest 2.3 [58]. The resulting best-fit model for each of 12 genes was then used for Bayesian analysis. Bayesian posterior probability values (BPP), shown above the nodes, were estimated after the initial one-third of the trees was discarded as burn-in (see methods for analysis details).

Phylogenetic relationships among major groups of nematodes represented by 36 complete mitochondrial genomes were inferred using two different tree-building methods for nucleotide and amino acid sequence datasets for the 12 protein-coding genes common to all the species. The concatenated dataset for nucleotide and amino acid sequences comprised 11,727 and 3,909 aligned characters, respectively. In addition, the nucleotide dataset was analyzed after excluding 3^rd ^codon positions (7,818 characters). This was performed to assess the possible impact of third codon position saturation on reconstructing mtDNA phylogeny, particularly at deeper levels (Figures 1, 2, 3 and 4). Overall, relationships inferred from Bayesian analysis of amino acids differed in several respects from the analyses of nucleotides, with the AA tree recovering somewhat fewer clades consistent with classical taxonomy. Relationships among taxa within each sampled superfamily were in broad agreement irrespective of different data types (nucleotide versus amino acid sequences) and different analytic methods, with relatively minor topology differences (Figures 1, 2, 3 and 4) that are discussed subsequently. Considering orders represented by two or more taxa, Oxyurida, Spirurida, and Mermithida were each monophyletic in all analyses with very high nodal support. Ascaridida was monophyletic in three of four analyses (not in Bayesian analysis of AA data), and Rhabditida was not monophyletic in any analysis. Representatives of Enoplea were monophyletic in three of four analyses (not in ML analysis of all nt data). Chromadorean species were a monophyletic group with very high nodal support in all analyses. Relationships among certain enoplean species differed between these datasets and methods. In ML analysis of all nucleotide data (Figure 1), enopleans were not monophyletic, with a dorylaimid (*Xiphinema americanum*), mermithids, and a trichocephalid (*Trichinella spiralis*), each forming successive sister groups to the chromadoreans. In other analyses (Figures 2, 3 and 4), *X. americanum *was more closely related to other enopleans, but with unreliable nodal support in some cases. The unstable position of *X. americanum *may be due to its long branch length (not shown) relative to other enoplean taxa. Reversals of strand-specific nucleotide bias in mtDNA can lead to convergences in sequence composition and long branch artifacts that can mislead phylogenetic analyses [30,31]. For nematode mitochondrial genomes, differences in patterns of strand-specific bias among species are only beginning to be investigated, but it is known that the mtDNA molecules of Chromadorean species have relatively conserved synteny and genes that are encoded on the same strand, whereas genes of Enoplean species are encoded by both strands and show much greater variation in organization [32]. The use of both DNA strands to encode genes may be more conducive to producing convergence through mutational constraints and introduction of potential bias affecting phylogenetic analyses [30].

Some chromadorean taxa also showed variation in phylogenetic position among analyses and between datasets. Some of these differences likely reflect differences in potential resolution of different datasets (e.g., amino acid characters versus nucleotide data). For example, relationships within superfamilies of Rhabditida often received higher ML bootstrap values in analyses of all nucleotides than the dataset that excluded 3^rd ^positions of codons. ML and BI analyses of the nucleotide datasets recovered all Ascaridida (including *C. robustus*) as monophyletic (99% BP), whereas in the BI of amino acid data (Figure 2), *C. robustus *(Seuratoidea) was sister (with high posterior probability; 0.99 BPP) to a group of Ascaridida and Rhabditida. Notably, one molecular phylogeny based on nuclear SSU rDNA also yielded an unexpected relationship for a different seuratoid genus, indicating more recent common ancestry with members of Rhabditida and Diplogasterida [10]. Similarly, although the position of *Steinernema carpocapsae *(an entomopathogenic nematode used in biological control) was strongly supported in Bayesian analysis of amino acids (Figure 2), it was unstable and poorly supported in analyses of nucleotide data. *Steinernema *has also been of unstable or conflicting phylogenetic position in analyses of SSU and LSU nuclear ribosomal DNA [7,11,33]. In some recent classifications reflecting certain SSU trees [6], *Steinernema *and *Strongyloides *have been placed in the same superfamily (Strongyloidoidea), and this taxonomic convention was followed in this study. However, *Steinernema *plus *Strongyloides *was never recovered as monophyletic in analyses of mitochondrial genome data, and instead *Steinernema *was more closely related to other Rhabditida plus Ascaridida (Figures 1, 3 and 4). Analysis of nuclear LSU rDNA also depicts *Steinernema *as more closely related to Rhabditida such as *Caenorhabditis *and *Heterorhabditis *than to *Strongyloides *[11]. Although some studies based on nuclear SSU rDNA have depicted *Steinernema *as sister to *Strongyloides *and closely related to free living Panagrolaimoidea [7,8], other studies have found the position of this genus to be unresolved by SSU sequences [33]. Perhaps factors influencing the unstable phylogenetic position of *Steinernema *are common to ribosomal DNA and mtDNA genes, and resolving the position of this important entomopathogenic genus will require sequences from other loci.

Certain older classifications emphasizing vertebrate parasites [34,35] have included *Strongyloides *within Rhabditoidea. In analyses of mtDNA genome data, *Strongyloides stercoralis *was recovered as the sister taxon to the Ascaridida+Rhabditida clade with very robust nodal support in all analyses (Figures 1, 2, 3 and 4). This position of *S. stercoralis *does not conflict with results obtained from analysis of nuclear ribosomal DNA, although trees inferred from LSU rDNA showed instability of topology that may result from compositional bias or long-branch attraction for *S. stercoralis *[11]. It is interesting to note that the gene order of *S. stercoralis *mtDNA is unique, differing substantially from those of other rhabditid taxa whose complete sequence has been reported so far [19]. Additional complete mitochondrial genome sequences of rhabditids, including species from the suborder Cephalobina (e.g., Panagrolaimoidea, Cephaloboidea) will be needed to assess if phylogenies based on mitochondrial genes are consistent with hypotheses obtained from nuclear genes, and to better understand mitochondrial genome evolution within Chromadorea. Representation of mitochondrial genomes from as yet unsampled clades (e.g., Diplogasterida, Tylenchida) may prove important for stabilizing or modifying relationships in parts of the mitochondrial gene tree; taxa that break long branches are believed to be particularly valuable additions.

Of eight superfamiles within Chromadorea represented by two or more species, five were monophyletic in all analyses (Trichostronyloidea, Rhabditoidea, Ascaridoidea, Oxyuroidea, and Filarioidea). Strongyloidoidea and Ancylostomatoidea (hookworms), were not monophyletic in any analysis, whereas Strongyloidea was monophyletic in three of four analyses (not in Bayesian analysis of AA data; Figure 2). Relationships among the five filarioid species were identical among the different inference methods and datasets, and these groups received strong nodal support. Tree topology for relationships among clade III orders was not consistent among the analyses, but in no case was clade III monophyletic. In addition, the four Ascaridida species were nested within Rhabitida in all analyses. Comparison of the best tree for all nucleotide data (Figure 1) with the optimal ML tree for this dataset constrained for clade III monophyly (tree topology provided in Methods), indicated that monophyly of clade III was a significantly worse interpretation of these data (Shimodaira-Hasegawa test, P < 0.05). Similarly, the S-H test revealed that the best ML tree consistent with clade III monophyly for the dataset excluding 3^rd ^positions was significantly worse (P < 0.05) than the best ML tree (Figure 3) for these data. The deepest node including all Rhabditida and Ascaridida had 100% bootstrap support (ML analyses) or 1.00 posterior probability (Bayesian analyses). In three of four analyses (Figures 1, 2 and 3), oxyurids were sister to Ascaridida plus Rhabditida but with strong nodal support only for analyses of the amino acid dataset. In the Bayesian analysis of nucleotide data excluding 3^rd ^positions (Figure 4), oxyurids were sister to spirurids, but with low posterior probability (0.83 BPP). This difference in position of the oxyurids, was the most marked difference between the analyses of complete data (nt or AA) versus nucleotide data excluding 3^rd ^positions. An alternative tree topology test based on ML and the dataset excluding 3^rd ^positions showed that the best ML tree constrained to require a sister taxon relationship between oxyurids and spirurids was significantly worse (S-H test, P < 0.05). This result confirms that there is a significant difference between inference methods (ML versus Bayesian) for the same dataset (nt excluding 3^rd ^positions) when considering the position of oxyurids. Although one analysis (Bayesian AA; Figure 2) showed strong nodal support for higher-level relationships involving oxyurids, a conservative interpretation is that additional investigations are required to confirm the relationship of oxyurids among Chromadorea based on mtDNA genomes. Phylogenetic analyses of SSU sequences did not provide reliable support for the relationship of oxyurids to other nematode taxa [10], suggesting that data from other loci may be necessary to resolve this question.

Nematodes in the orders Ascaridida, Oxyurida, and Spirurida are a major component of zooparasitic diversity. Evolutionary hypotheses and classification schemes for these orders have differed substantially, depending on the types of data considered and the taxonomic authority. For example, Chitwood [36] and Maggenti [37] both suggested that Ascaridida was more closely related to Rhabditida than to Spirurida. Specifically, Maggenti's [38] classification placed special emphasis on the morphology of the esophagus and life-history traits, assigning the orders Rhabditida, Strongylida and Ascaridida under the subclass Rhabditia. In contrast, other classifications emphasizing different features suggested a sister-group relationship between Ascaridida and Spirurida [35,39], and proposed that members of this clade are more closely related to Strongylida than to Oxyurida and Rhigonematida. In other work based on traditional approaches, Inglis [40,41] proposed a sister-group relationship between Oxyurida and Strongylida, with Ascaridida and Spirurida belonging to a separate clade. These hypotheses predate the understanding, inferred from molecular phylogenetics [7,8], that the vertebrate parasites of the classical order Strongylida are phylogenetically nested within and closely related to free-living Rhabditoidea. Phylum-wide phylogenies based on SSU rDNA have indicated that representatives of the animal-parasitic orders Ascaridida, Spirurida, Rhigonematida and Oxyurida form a monophyletic group with strong support (clade III *sensu *[7]; [10][42]), with the exception of a single species in one study [10]. In contrast to this overall monophyly for clade III taxa, an analysis of SSU sequences for > 100 clade III species revealed that three of the component orders (Ascaridida, Spirurida, and Rhigonematida) each lacked monophyly for their sampled species [10]. Finally, SSU data provide inconclusive resolution for relationships among most major clade III lineages [10]. For clade III taxa, mitochondrial genome data is inconsistent with clade III monophyly, and instead depicts Ascaridida as nested within Rhabditida. The mitochondrial phylogenetic hypothesis conflicts with the evolutionary proposals by Inglis [40] and Anderson [35,39], but is more consistent with the proposal of Maggenti [37,38] in that the Rhabditida, Strongylida, and Ascaridida are more closely related to each other than any of these orders are to Spirurida. Maggenti [37] also treated oxyurids as a superfamily within Ascaridida, but our mtDNA trees do not support a close relationship between ascaridids and oxyurids. Previous results based on substantially fewer mitochondrial genome sequences are consistent with the current conclusions based on broader taxon sampling, for example, support for a sister-group relationship between Ascaridida and Rhabditida [20] and absence of monophyly for representative clade III species [21]. In the context of mtDNA phylogenetics, characterizing the relationships among parasitic nematodes and understanding their relationships to free-living species will require sampling of many additional lineages, which is becoming feasible with high-throughput sequencing methods. However, the current mtDNA genome phylogeny is not consistent with an evolutionary hypothesis suggesting two separate origins of zooparasitism, one for Ascaridida and Spirurida and a separate origin for Oxyurida and Strongylida (Strongyloidea) [41].

**Figure 5:**
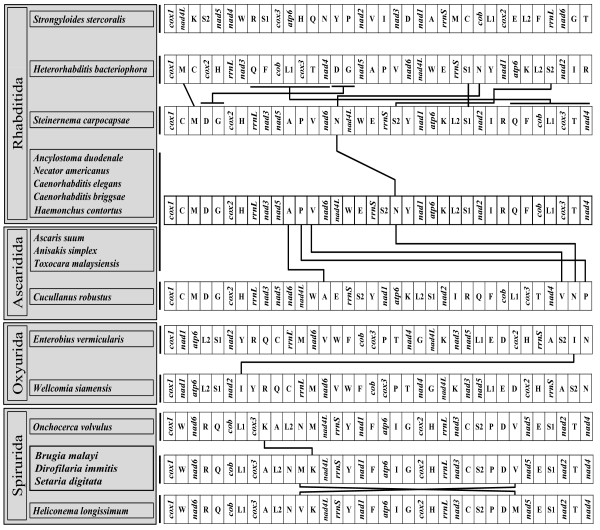
**Linearized representation of the mitochondrial gene arrangement for representatives of major chromadorean nematode clades, including three species newly determined in this study**. Gene and genome size are not to scale. All genes are transcribed in the same direction (from left to right). The tRNAs are designated by single-letter abbreviation and two leucine and two serine tRNA genes are labeled, according to their anticodon sequence, as L1 (*trnL-uag*), L2 (*trnL-uaa*), S1 (*trnS-ucu*), and S2 (*trnS-uga*), respectively.

In general, the gene content of mitochondrial genomes is invariant with few changes across a variety of metazoans. Based on the viewpoint that gene order is also rather stable, and that gene rearrangements tend to reflect common ancestry rather than convergent evolution, comparison of mitochondrial gene order pattern has often been suggested as a potential tool for resolving deep phylogenetic relationships [43-45]. Yet, these assumptions have been called into question as more mitochondrial genome data has been obtained. There is a growing body of evidence that extensive gene rearrangements have occurred within some metazoan groups including enoplean nematodes [46], mollusks [47,48], tunicates [49], and some crustacean arthropods [50]. Such exceptions challenge the utility of gene order for inferring deep branch phylogeny. In this context, comparison of gene order does not appear useful for evaluating relationships among enoplean nematodes because extensive gene rearrangement is known even among closely related taxa [46]. In contrast, mitochondrial gene order of chromadorean nematode species appears to be more conserved, and shared gene arrangement between the taxa has been interpreted as a reliable indicator of their phylogenetic affinity [20,21]. Considering the phylogenetic framework provided by analysis of mtDNA genome sequences, a highly conserved gene order is found among most members within the Ascaridida+Rhabditida clade. For *C. robustus*, the newly determined genome sequence has a gene arrangement that is almost identical to those of most Ascaridida and Rhabditida, with only a few translocations of some tRNAs (*trnA, trnV, trnN *and *trnP*) (Figure 5). The notable exceptions to highly conserved gene arrangement within the Ascaridida+Rhabditida clade are found in *Strongyloides stercoralis *and *Heterorhabditis bacteriophora *whose gene arrangements differ substantially from all other members of the clade reported thus far; the former species is the only representative of Strongyloidoidea in the analysis, and *S. stercoralis *is consistently placed as the sister lineage to the other Ascaridida+Rhabditida. Gene arrangement among the species sampled within each of the orders Oxyurida and Spirurida is very similar and translocations within each order are very limited (Figure 5). For example, there is a single translocation or reciprocal translocation of a tRNA in Oxyurida (*trnI *between *Enterobius vermicularis *and *Wellcomia siamensis*) and reciprocal translocations of *trnV *and *trnM *between *Heliconema longissimum *and *Dirofilaria immitis-Brugia malayi-Setaria digitata *(Spirurida), and a single translocation of *trnK *between *Onchocerca volvulus *and *Dirofilaria immitis-Brugia malayi-Setaria digitata *(Spirurida). Despite the similarity within most orders (excepting Rhabditida), notable differences in gene arrangement pattern are found among different orders, particularly between Oxyurida and Spirurida and/or between Spirurida and most Ascaridida and Rhabditida members. Although the gene arrangement of oxyurid members sequenced to date is unique, there are some partial gene order identities (*nad1-atp6, trnL2-trnS1-nad2, trnR-trnQ, trnT-nad4*, and *nad3-nad5*) shared among most members of Rhabditida, Ascaridida and Oxyurida (Figure 5). In contrast, these identities are not shared with Spirurida species. Although interpretation of gene order patterns would benefit from formal phylogenetic analysis of these data, this has not yet been performed because additional sampling of more chromadorean groups is needed, particularly since the high variability in gene order among enopleans complicates their utility for rooting the chromadorean tree. Nevertheless, shared gene arrangement patterns appear to provide additional support for the hypothesis that Spirurida are not closely related to Oxyurida and Ascaridida, and that the latter two groups (especially Ascaridida) share a more recent common ancestor with Rhabditida. Neither mitochondrial genome phylogenies nor gene arrangement patterns support the hypothesis that clade III nematodes (*sensu *[7]) share a most recent common ancestor reflecting a single origin.

## Conclusion

In this study, we tested phylogenetic relationships of nematodes using complete mitochondrial genome information, with special emphasis on species from clade III, which includes major components of zooparasitic nematode diversity. For this study, we determined the complete mitochondrial genome sequences of three nematode species, *Cucullanus robustus *(13,972 bp) from Ascaridida, *Heliconema longissimum *(13,610 bp) from Spirurida, and *Wellcomia siamensis *(14,128 bp) from Oxyurida, and used sequences from the protein coding genes to investigate phylogenetic relationships, focusing on chromadorean orders. Phylogenetic analysis of these new mitochondrial genomes corroborates the close relationship between Ascaridida and Rhabditida and confirms that Ascaridida is nested within Rhabditida. The Oxyurida and Spirurida are each monophyletic, and are sister groups to the Ascaridida plus Rhabditida clade. This lack of monophyly for clade III, as inferred from mtDNA genomes, conflicts with phylogenetic trees based on nuclear rDNA. These mtDNA hypotheses suggest that zooparasitic nematodes represented by Ascaridida, Oxyurida and Spirurida do not share a most recent common ancestor. Ultimately, resolution of this and other conflicts between nuclear rDNA gene trees and mtDNA gene trees will require hypotheses based on additional independent loci. However, regions of topological concordance between mtDNA and nuclear rDNA phylogenetic hypothesis indicate that continued efforts to obtain complete mitochondrial genomes for unrepresented nematode lineages will prove useful for understanding the evolution of mtDNA genomes and developing phylogenetic hypotheses for the phylum Nematoda.

## Methods

### Sample collection and molecular techniques

Nematode specimens were isolated from their host animals (*C. robustus *from the conger eel *Conger myriaster*, *H. longissimum *from the Japanese eel *Anguilla japonica*, and *W. siamensis *from the Malayan porcupine *Hystrix brachyura*) and kept in 70% ethanol until they were used for total genomic DNA extraction. Total genomic DNA was extracted using a commercial kit (Qiagen Co.) according to the manufacturer's protocol. Partial fragments from different regions of the mitochondrial genome were initially amplified and directly sequenced using universal primer combinations or primer sets designed directly from the conserved regions of nematode mitochondrial gene sequences ([51]; see Additional file 4 for primer details). Partial fragments of *cox1*, *rrnS *and *rrnL *were amplified for *C. robustus*; partial fragments of *rrnS*, *rrnL*, *cob *and *nad4 *were amplified for *H. longissimum*, and partial fragments of *rrnS*, *rrnL*, *cox1 *and *nad4 *were amplified for *W. siamensis*. PCR reactions were carried out in a 50 μl reaction volume consisting of 10 units of *Taq *polymerase (Roche), 2.5 mM dNTP mixture, 2.5 mM MgCl_2_, and 20 pmole of each primer with the following amplification conditions: one cycle of the initial denaturation step at 95°C for 2 min, followed by 35 cycles of denaturation at 95°C for 30 s, primer annealing at 43~48°C for 30 s and elongation at 72°C for 1 min. The final step was extension at 72°C for 10 min.

The nucleotide sequences obtained from each of these gene fragments were then used to design species-specific primer sets for long PCR reactions. These overlapping long PCR products (ranging from ~1.5 kb to 8 kb in size), covering the entire mitochondrial genome, were amplified using the long PCR primer sets (Additional file 4) and the Expand Long Template PCR System (Roche) with the following amplification conditions: 1 cycle of initial denaturation (2 min at 93°C), 30 cycles of denaturation - primer annealing - elongation (15 s at 93°C, 30 s at 50~60°C, and 10 min at 68°C), and 1 cycle of the final extension (10 min at 68°C). The amplified long PCR products were gel-isolated, and extracted using the TOPO Gel Purification reagents supplied with the TOPO XL cloning kit (Invitrogen Co.). After gel purification, each of the long PCR products was ligated using the TOPO XL cloning kit and then transformed into competent *E. coli*. Cycle sequencing reactions for each of the long PCR products were performed in both directions by the 'primer walking' method using a Big Dye Terminator Cycle-Sequencing Kit (Applied Biosystems). A complete strand of the entire mtDNA sequence from each of the three species was then assembled by double-checking the sequences of overlapping regions of the long PCR fragments and partial sequences obtained from separate gene fragments.

### Gene annotation and phylogenetic analyses

Twelve mitochondrial protein-coding genes and two ribosomal RNA genes of each species were identified by finding gene boundaries based on comparison with other nematode mitochondrial DNA sequences. Putative secondary structures of 22 tRNA genes from each of the three mtDNAs were identified using the tRNAscan-SE program [52] or by manually finding potential secondary structures and anticodon sequences. Thirty-six complete nematode mitochondrial genomes including three newly sequenced in the present study were used for phylogenetic analysis with two arthropod species (*Lithobius forficatus *and *Limulus polyphemus*) as outgroups. A complete list of species, their taxonomy and GenBank accession numbers are provided in Additional file 5. For phylogenetic analysis, both nucleotide and amino acid sequence datasets from the 12 protein-coding genes were used. For multiple alignment of amino acid sequences, the nucleotide sequences of each of 12 protein-coding genes were first translated into amino acids using the invertebrate mitochondrial genetic code. The resulting amino acid sequences were then aligned for each gene using Clustal × with default options [53]. The nucleotide sequences of the 12 protein-coding genes were aligned based on the framework of their corresponding amino acid alignment using RevTrans, a web-based program for placing gaps in coding DNA based on amino acid alignments [54]. Alignments of individual genes were concatenated for phylogenetic analysis. A nucleotide dataset excluding 3^rd ^positions of codons was also constructed. Phylogenetic analyses for the concatenated datasets (full nucleotide, nucleotide excluding 3^rd ^positions, amino acids) were performed using two different tree-building methods. Bayesian inference was used for the amino acid dataset and conducted using the codon model for MrBayes version 3.1.2 [55]. MrBayes was run using four MCMC chains for 10^6 ^generations, and sampled every 1,000 generations. Each of the 12 genes was treated as a separate unlinked data partition. For the amino acid dataset, Bayesian posterior probability (BPP) values were determined after discarding the initial 200 trees (the first 2 × 10^5 ^generations) as burn-in. With the nucleotide dataset excluding 3^rd ^positions, each of the 12 genes was treated as a separate unlinked data partition. MrBayes was executed on the Cipres Portal for the nucleotide datasets and using four MCMC chains for 4 × 10^6 ^generations, sampled every 4,000 generations. Bayesian posterior probability (BPP) values were determined after discarding the initial one-third of trees as burn-in. Maximum likelihood analysis was used for the two nucleotide datasets and conducted using RAxML 7.0.3 [56] and the CIPRES web portal. For RAxML, each of the 12 genes was treated as a separate partition (with gamma rate heterogeneity and all gamma model parameters estimated for each partition by the program). Bootstrap ML analysis was performed using the rapid bootstrapping method (RAxML) with 1,000 replicates. Statistical tests for comparing alternative phylogenetic hypotheses were performed using both the complete nucleotide dataset and the dataset excluding 3^rd ^positions of codons; alternative trees were evaluated using the likelihood-based Shimodaira-Hasegawa test [57] as implemented in RAxML 7.0.3. Alternative trees for comparison were found based on RAxML searches (with gene partitions as detailed previously), but with the tree topology constrained to reflect the alternative hypothesis of choice (e.g., constrained for clade III monophyly).

The best ML tree recovered for the complete nt dataset constrained for clade III monophyly was: [((*Lithobius forficatus *,((*Strongyloides stercoralis*,(*Steinernema carpocapsae*,((((*Enterobius vermicularis*, *Wellcomia siamensis*),(*Heliconema longisimum*,(*Setaria digitata*,(*Brugia malayi*,(*Dirofilaria immitis*, *Onchocerca volvulus*))))),(*Cucullanus robustus*,((*Anisakis simplex*, *Toxocara malaysiensis*), *Ascaris sum*))),(((((*Trichostrongylus axei*, *Cooperia oncophora*),((*Mecistocirrus digitatus*, *Haemonchus contortus*), *Teladorsagia circumcincta*)),(*Necator americanus*,((*Ancylostoma duodenale*, *Ancylostoma caninum*),((*Syngamus trachea*, *Strongylus vulgaris*),(*Chabertia ovina*, *Oesophagostomum dentatum*))))),*Metastrongylus pudendotectus*),(*Heterorhabditis bacteriophora*,(*Caenorhabditis elegans*, *Caenorhabditis briggsae*)))))),(((((*Agamermis sp*., *Hexamermis agrotis*),(*Romanomermis culicivorax*, *Strelkovimermis spiculatus*)), *Thaumamermis cosgrovei*), *Xiphinema americanum*), *Trichinella spiralis*))), *Limulus polyphemus*);].

The best ML tree recovered for the dataset excluding 3^rd ^positions and constrained for clade III monophyly was: [((*Lithobius forficatus*,((*Trichinella spiralis*,((*Thaumamermis cosgrovei*,((*Agamermis *sp., *Hexamermis agrotis*),(*Romanomermis culicivorax*, *Strelkovimermis spiculatus*))), *Xiphinema americanum*)),(*Strongyloides stercoralis*,(*Steinernema carpocapsae*,((((*Enterobius vermicularis*, *Wellcomia siamensis*),((((*Onchocerca volvulus*, *Dirofilaria immitis*), *Brugia malayi*), *Setaria digitata*), *Heliconema longisimum*)),(*Cucullanus robustus*,(*Anisakis simplex*,(*Ascaris sum*, *Toxocara malaysiensis*)))),(((((*Ancylostoma caninum*, *Ancylostoma duodenale*),((*Chabertia ovina*, *Oesophagostomum dentatum*),(*Syngamus trachea*, *Strongylus vulgaris*))), *Necator americanus*),(*Metastrongylus pudendotectus*,(*Trichostrongylus axei*,(( *Cooperia oncophora*, *Teladorsagia circumcincta*),(*Haemonchus contortus*, *Mecistocirrus digitatus*))))),(*Heterorhabditis bacteriophora*,(*Caenorhabditis briggsae*, *Caenorhabditis elegans*)))))))), *Limulus polyphemus*);].

## Abbreviations

AA: amino acid; *atp6*, and *atp8*: genes for ATP synthase subunits 6 and 8; BI: Bayesian inference; bp: base pair; BP: bootstrap percentage; BPP: Bayesian posterior probability; *cob*: gene for cytochrome oxidase *b*; *cox1*-*cox3*: genes for cytochrome oxidase *c *subunit 1-3; dNTP: deoxyribonucleotide triphosphate; kb: kilo base; LSU: large subunit nuclear ribosomal DNA; ML: maximum likelihood; mtDNA: mitochondrial DNA; *nad1-6*, and *nad4L*: genes for NADH dehydrogenase subunits 1-6 and 4L; NCR: non-coding region; nt: nucleotide; PCR: polymerase chain reaction; *rrnS*, and *rrnL*: genes for small and large mitochondrial ribosomal RNA subunits; SSU: small subunit nuclear ribosomal DNA; tRNA: transfer RNA.

## Authors' contributions

JKP designed the study, performed phylogenetic analyses, interpreted the results and drafted the manuscript. TS, SHL, SK, HKK, and GSM participated in laboratory work for mitochondrial genome sequencing. KSE and SAN also participated in phylogenetic analyses, interpretation of trees, and drafting the manuscript. All authors read and approved the final manuscript.

## Supplementary Material

Additional File 1 **Circular gene maps of the complete mitochondrial genome for *Cucullanus robustus *(A), *Wellcomia siamensis *(B), and *Heliconema longissimum *(C)**. All genes are encoded in the same direction and 22 tRNA genes are designated by a single-letter abbreviation. The two leucine and two serine tRNA genes are labeled, according to their anticodon sequence, as L1 (*trnL-uag*), L2 (*trnL-uaa*), S1 (*trnS-ucu*), and S2 (*trnS-uga*), respectively. Click here for file

Additional File 2 **Nucleotide composition of mitochondrial genomes of *Cucullanus robustus, Wellcomia siamensis *and *Heliconema longissimum***. Click here for file

Additional File 3 **The predicted secondary structures of 22 tRNAs for the three species with complete mtDNA sequences determined in this study**. (A) *Cucullanus robustus*, (B) *Wellcomia siamensis*, and (C) *Heliconema longissimum*. Click here for file

Additional File 4 **The PCR primer information used in this study**. (A) *Cucullanus robustus*, (B) *Wellcomia siamensis*, and (C) *Heliconema longissimum*. Click here for file

Additional File 5 **The species, taxonomy, and GenBank accession numbers for nematode species used in phylogenetic analyses in this study**. Click here for file
