# Supplementary figures and images for: Monophyly of clade III nematodes is not supported by phylogenetic analysis of complete mitochondrial genome sequences
Source: BMC Genomics. 2011 Aug 3;12:392. doi: 10.1186/1471-2164-12-392 (PMC3163570; doi:10.1186/1471-2164-12-392)

## Additional file 1A

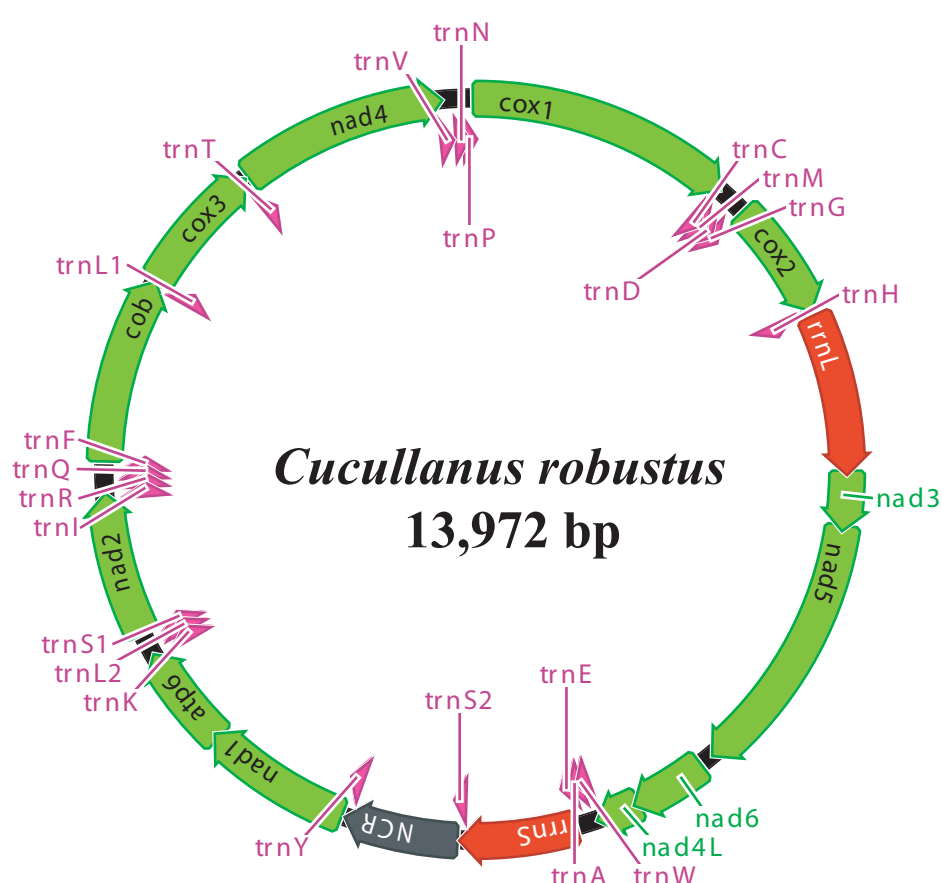

## Additional file 1B

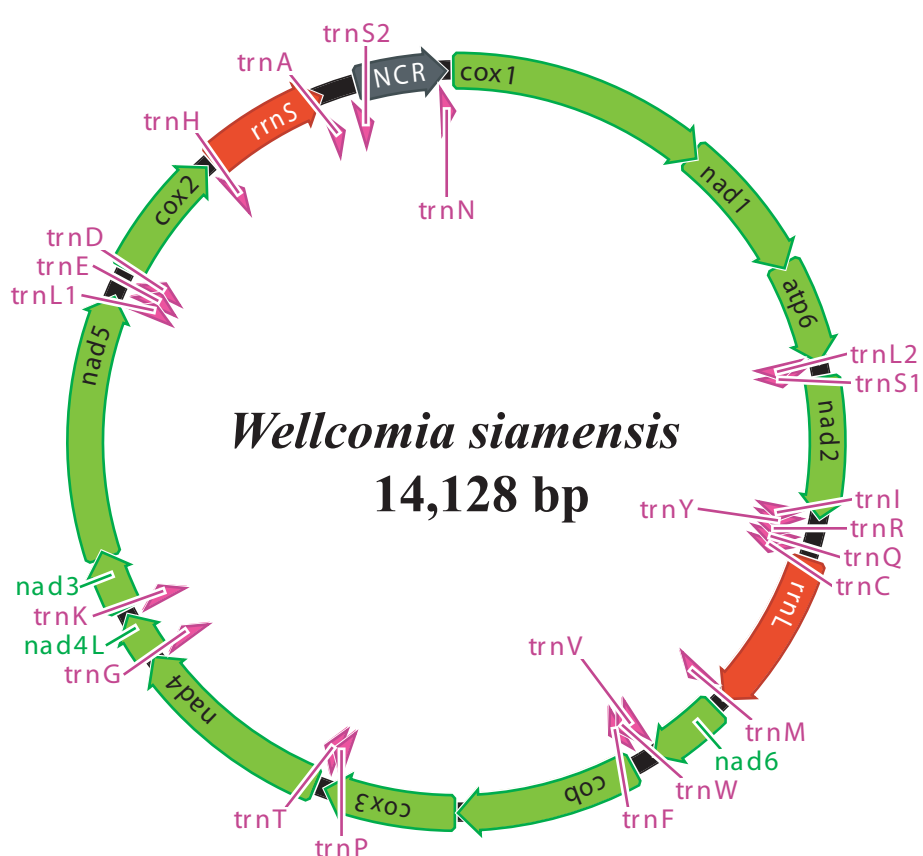

### Additional file 1C

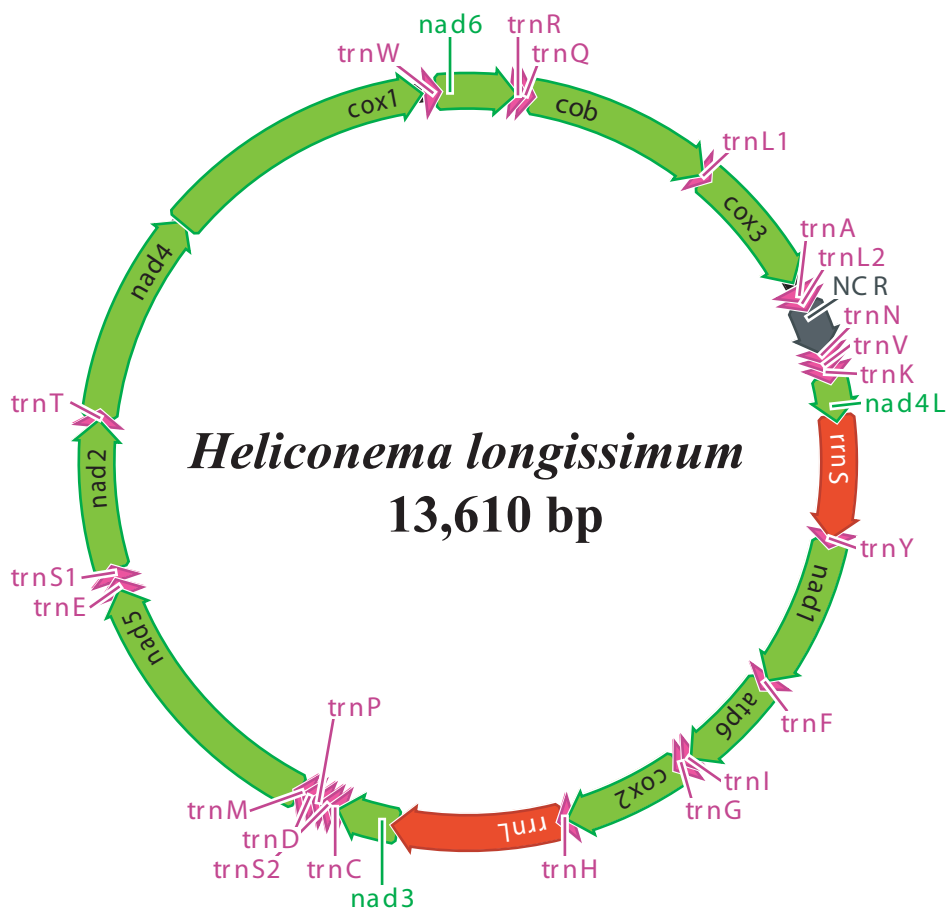

Supplement: Additional File 1 — Circular gene maps of the complete mitochondrial genome for Cucullanus robustus (A), Wellcomia siamensis (B), and Heliconema longissimum (C). All genes are encoded in the same direction and 22 tRNA genes are designated by a single-letter abbreviation. The two leucine and two serine tRNA genes are labeled, according to their anticodon sequence, as L1 (trnL-uag), L2 (trnL-uaa), S1 (trnS-ucu), and S2 (trnS-uga), respectively. [file 1471-2164-12-392-S1.PDF]
