# Supplementary material for: Monophyly of clade III nematodes is not supported by phylogenetic analysis of complete mitochondrial genome sequences
Source: BMC Genomics. 2011 Aug 3;12:392. doi: 10.1186/1471-2164-12-392 (PMC3163570; doi:10.1186/1471-2164-12-392)
Supplement: Additional File 2 — Nucleotide composition of mitochondrial genomes of Cucullanus robustus, Wellcomia siamensis and Heliconema longissimum. [file 1471-2164-12-392-S2.PDF]

Additional file 2. Codon usage for 12 protein coding genes of *Cucullanus robustus* (Cr), *Wellcomia siamensis* (Ws), *Heliconema longissimum* (Hl) mtDNAs

Codon	AA	No.			%			Codon	AA	No.			%		
		Cr	Ws	Hl	Cr	Ws	Hl			Cr	Ws	Hl	Cr	Ws	Hl
TTT	Phe	447	643	614	13.10	18.88	18.00	TAT	Tyr	140	171	205	4.10	5.02	6.01
TTC	Phe	43	2	12	1.26	0.06	0.35	TAC	Tyr	18	1	9	0.53	0.03	0.26
TTA	Leu	236	299	321	6.91	8.78	9.41	TAA	*	0	0	0	0.00	0.00	0.00
TTG	Leu	224	207	117	6.56	6.08	3.43	TAG	*	0	0	0	0.00	0.00	0.00
CTT	Leu	67	11	16	1.96	0.32	0.47	CAT	His	46	52	52	1.35	1.53	1.52
CTC	Leu	8	0	0	0.23	0.00	0.00	CAC	His	6	0	1	0.18	0.00	0.03
CTA	Leu	24	1	11	0.70	0.03	0.32	CAA	Gln	10	18	27	0.29	0.53	0.79
CTG	Leu	18	1	2	0.53	0.03	0.06	CAG	Gln	29	18	17	0.85	0.53	0.50
ATT	Ile	195	257	301	5.71	7.55	8.82	AAT	Asn	98	119	124	2.87	3.49	3.64
ATC	Ile	35	0	6	1.03	0.00	0.18	AAC	Asn	16	0	1	0.47	0.00	0.03
ATA	Met	98	84	145	2.87	2.47	4.25	AAA	Lys	33	49	57	0.97	1.44	1.67
ATG	Met	81	68	33	2.37	2.00	0.97	AAG	Lys	57	36	20	1.67	1.06	0.59
GTT	Val	174	242	135	5.10	7.11	3.96	GAT	Asp	55	68	68	1.61	2.00	1.99
GTC	Val	31	3	5	0.91	0.09	0.15	GAC	Asp	13	1	3	0.38	0.03	0.09
GTA	Val	41	47	42	1.20	1.38	1.23	GAA	Glu	22	40	37	0.64	1.17	1.08
GTG	Val	74	40	12	2.17	1.17	0.35	GAG	Glu	54	30	26	1.58	0.88	0.76
TCT	Ser	153	98	126	4.48	2.88	3.69	TGT	Cys	48	77	71	1.41	2.26	2.08
TCC	Ser	4	2	10	0.12	0.06	0.29	TGC	Cys	3	0	3	0.09	0.00	0.09
TCA	Ser	25	11	16	0.73	0.32	0.47	TGA	Trp	26	49	46	0.76	1.44	1.35
TCG	Ser	10	5	1	0.29	0.15	0.03	TGG	Trp	52	24	40	1.52	0.70	1.17
CCT	Pro	51	54	64	1.49	1.59	1.88	CGT	Arg	22	35	42	0.64	1.03	1.23
CCC	Pro	7	2	5	0.21	0.06	0.15	CGC	Arg	1	0	2	0.03	0.00	0.06
CCA	Pro	9	4	13	0.26	0.12	0.38	CGA	Arg	2	0	5	0.06	0.00	0.15
CCG	Pro	7	2	4	0.21	0.06	0.12	CGG	Arg	8	1	5	0.23	0.03	0.15
ACT	Thr	65	48	75	1.90	1.41	2.20	AGT	Ser	74	123	100	2.17	3.61	2.93
ACC	Thr	11	1	9	0.32	0.03	0.26	AGC	Ser	12	1	1	0.35	0.03	0.03
ACA	Thr	16	3	6	0.47	0.09	0.18	AGA	Ser	44	53	51	1.29	1.56	1.50
ACG	Thr	12	4	1	0.35	0.12	0.03	AGG	Ser	63	24	13	1.85	0.70	0.38
GCT	Ala	45	51	55	1.32	1.50	1.61	GGT	Gly	115	125	128	3.37	3.67	3.75
GCC	Ala	16	2	9	0.47	0.06	0.26	GGC	Gly	10	4	8	0.29	0.12	0.23
GCA	Ala	5	2	11	0.15	0.06	0.32	GGA	Gly	14	50	43	0.41	1.47	1.26
GCG	Ala	16	4	1	0.47	0.12	0.03	GGG	Gly	74	38	28	2.17	1.12	0.82

*: termination codons were not included.
