# Supplementary material for: Monophyly of clade III nematodes is not supported by phylogenetic analysis of complete mitochondrial genome sequences
Source: BMC Genomics. 2011 Aug 3;12:392. doi: 10.1186/1471-2164-12-392 (PMC3163570; doi:10.1186/1471-2164-12-392)

Additional file 3A. *Cucullanus robustus*

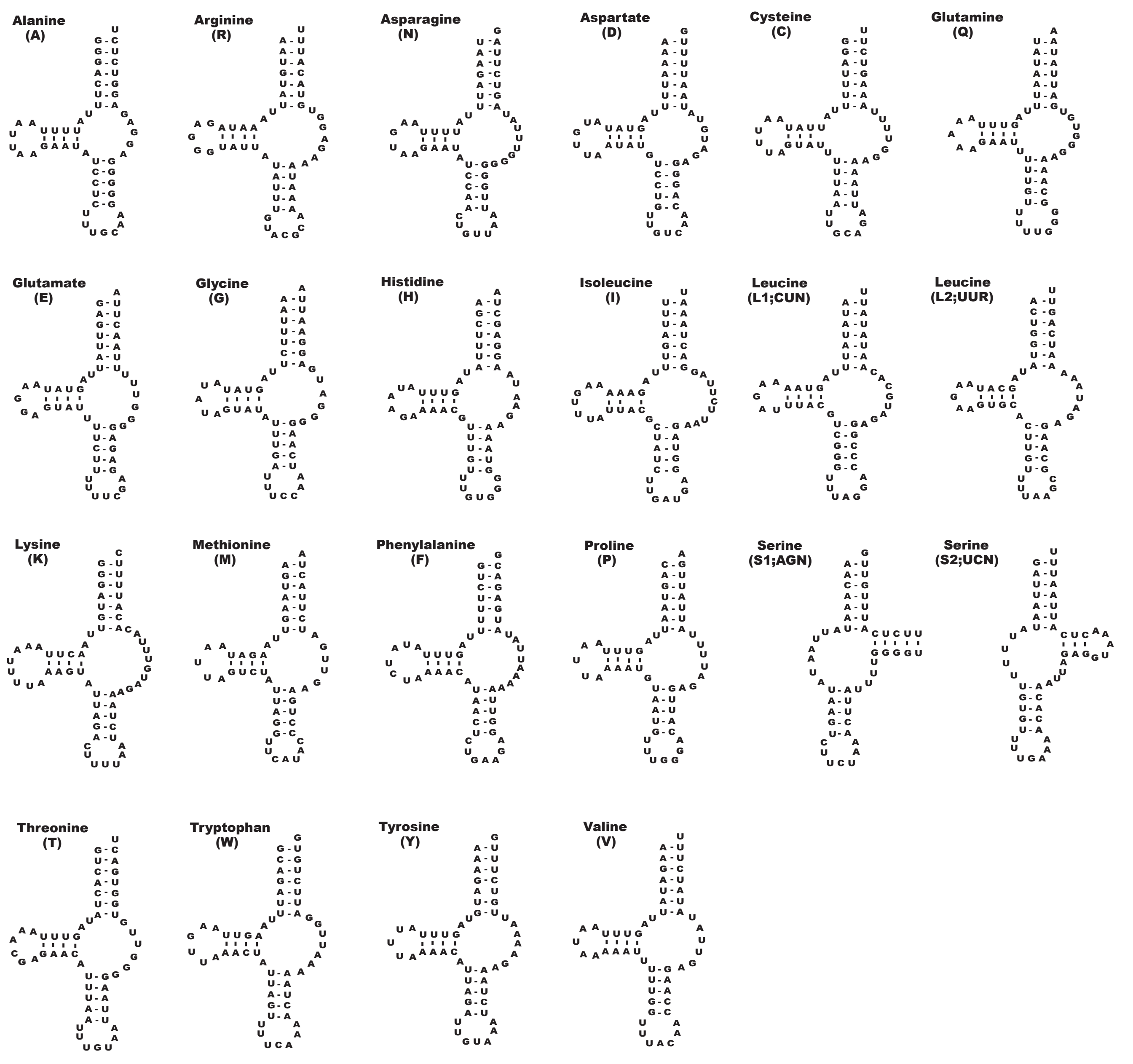

Additional file 3B. *Wellcomia siamensis*

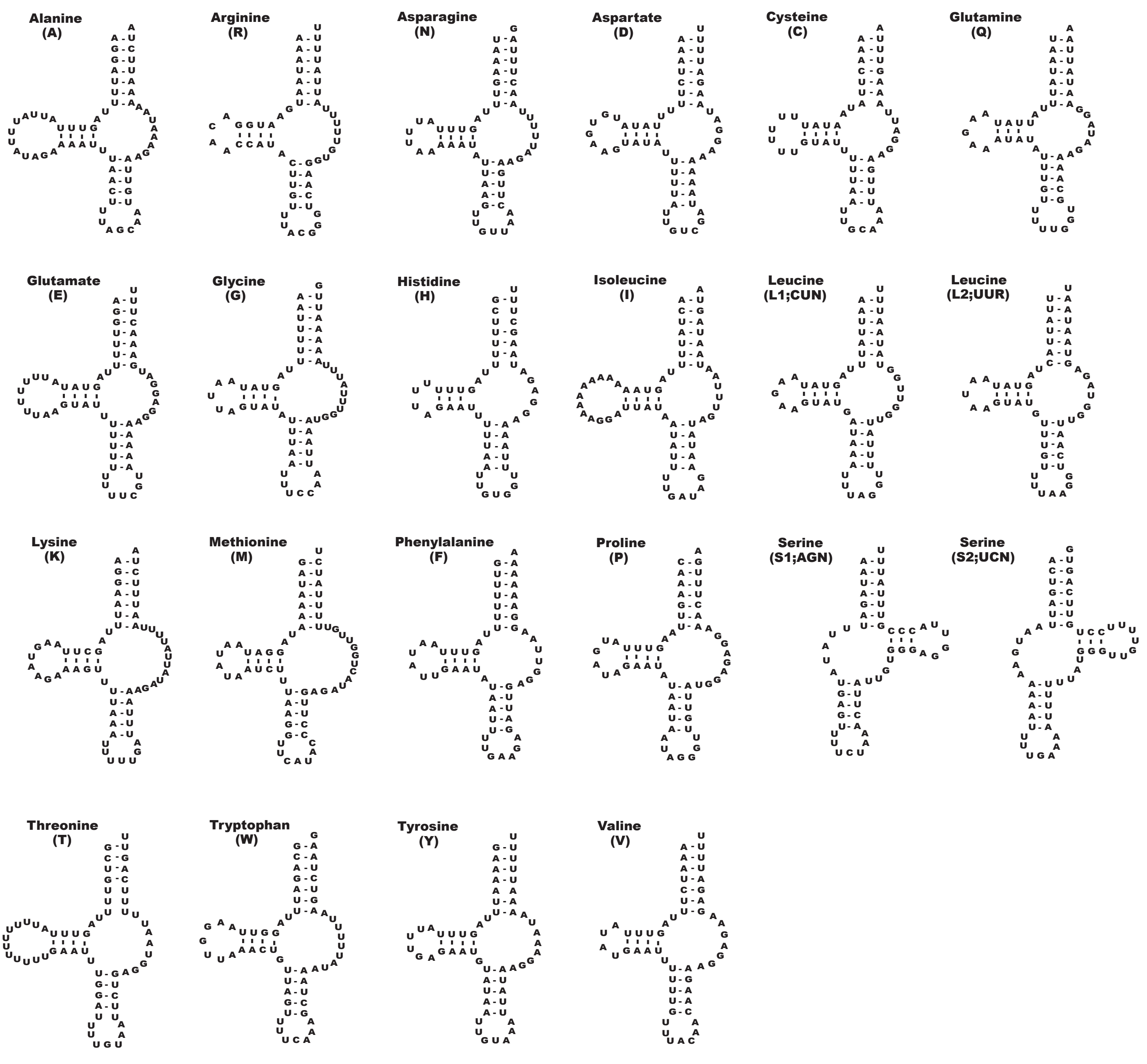

Additional file 3C. *Heliconema longissimum*

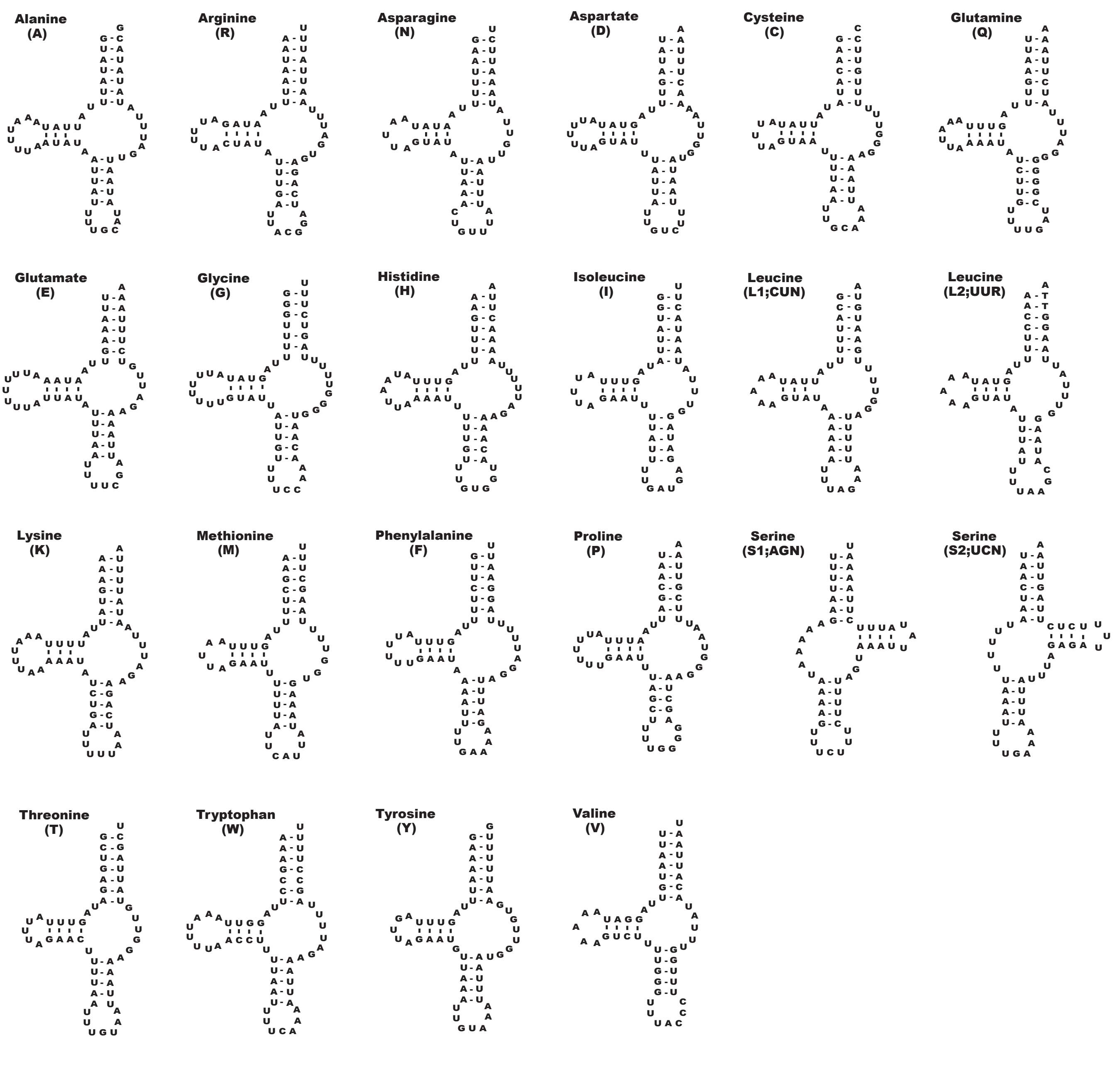

Supplement: Additional File 3 — The predicted secondary structures of 22 tRNAs for the three species with complete mtDNA sequences determined in this study. (A) Cucullanus robustus, (B) Wellcomia siamensis, and (C) Heliconema longissimum. [file 1471-2164-12-392-S3.PDF]
