# Supplementary material for: Monophyly of clade III nematodes is not supported by phylogenetic analysis of complete mitochondrial genome sequences
Source: BMC Genomics. 2011 Aug 3;12:392. doi: 10.1186/1471-2164-12-392 (PMC3163570; doi:10.1186/1471-2164-12-392)
Supplement: Additional File 4 — The PCR primer information used in this study. (A) Cucullanus robustus, (B) Wellcomia siamensis, and (C) Heliconema longissimum. [file 1471-2164-12-392-S4.PDF]

Additional file 4.

A: The PCR primers for *Cucullanus robustus* mtDNA used in this study

Primers	Sequence (5′–3′)	Source	Estimated size of PCR product
LCO1490<br>HCO2198	GGTCAACAACTCATAAAGATATTGG<br>TAAACTTCAGGGTGACCAAAAAATCA	[51]	~700 bp
Nema 12S-F<br>Nema 12S-R	GTTCC GAATAATC GCTA<br>GCKATTGARGGATGYTTTGTACC	This study	531bp
Nema-16S-F<br>Nema-16S-R	WWTAAATGGCAGYCTTAGCGTGA<br>TCTYMCRAYGAAYTAAACTAATATC	This study	443bp
Cuc-CO1-F<br>Cuc-16S-R	TTTAGGCTTCATTGTGCCGGGGTGAG<br>AGCATGATTTCCTGTTAACTCCGAAG	This study	2.5kb
Cuc-12S-F<br>Cuc-CO1-R	TGAAACGGATTAGTACCCGTGTAATCA<br>AATCTACCCTATTACCAGGATGACCTGTGG	This study	8kb
Cuc-16S-F<br>Cuc-12S-R	CTTCGGAGTTAACAGGAAATCATGC<br>TGATTACACGGGTACTAATCCGTTTCA	This study	3.5kb

B: The PCR primers for *Wellcomia siamensis* mtDNA used in this study

Primers	Sequence (5′–3′)	Source	Estimated size of PCR product
Nema 12S-F<br>Nema 12S-R	GTTCC GAATAATC GCTA<br>GCKATTGARGGATGYTTTGTACC	This study	531bp
Nema-16S-F<br>Nema-16S-R	WWTAAATGGCAGYCTTAGCGTGA<br>TCTYMCRAYGAAYTAAACTAATATC	This study	443bp
LCO1490<br>LCO2198	GGTCAACA AT ATAAA ATATTG G<br>TAAACTTCAGGGTGACC AAAAATCA	[51]	~700 bp
Nema-ND4-F<br>Nema-ND4-R	WWTAAATGGCAGYCTTAGCGTGA<br>TCTYMCRAYGAAYTAAACTAATATC	This study	443bp
Wel-12S-F<br>Wel-CO1-R	GAGAACCCTCATTTCTTACCGTTATTTTAG<br>AGATTCTCTTCATAAAATTCCAGGCTTTCC	This study	1.5kb
Wel-CO1-F<br>Wel-16S-R	TGTTGATAGGGGGGCGGGGACTGGA<br>ACATTCTGTTAACTCCGAAGTAATTTACAC	This study	4.5kb
Wel-16S-F<br>Wel-NAD4-R	TGTGTAAATTACTTCGGAGTTAACAGAATG<br>GAGCTCAACAAACGTAAAACTCCGACAGTG	This study	3.5kb
Wel-NAD4-F<br>Wel-12S-R	GCTCATGTGGAAGCTCCTACTGTGGCTAGG<br>CTAAAATAACGGTAAGAAATGAGGGTTCTC	This study	4.5kb

C: The PCR primers for *Heliconema longissimum* mtDNA used in this study

Primers	Sequence (5′–3′)	Source	Estimated size of PCR product
Nema 12S-F<br>Nema 12S-R	GTTCC GAATAATC GCTA<br>GCKATTGARGGATGYTTTGTACC	This study	531bp
Nema-16S-F<br>Nema-16S-R	WWTAAATGGCAGYCTTAGCGTGA<br>TCTYMCRAYGAAYTAAACTAATATC	This study	443bp
Spiru-CYTBF<br>Spiru-CYTBR	GCDGCDGTWGTTATTACTAGT<br>TTATCATAATCDCCATGACAATA	This study	204bp
Spiru-ND4F<br>Spiru-ND4R	TGRCTRCCTAAGGTTCATG<br>TAGCAATATGATAAAACTCACC	This study	360bp
Hel-12S-F<br>Hel-16S-R	GGAACATGTTTTGGAGAGTTCTCCTTACT<br>CTACTTACTCTGTTAACTCCGGAGTAAC	This study	3.5kb
Hel-16S-F<br>Hel-ND4-R	GTTACTCCGGAGTTAACAGAGTAAGTAGT<br>CAAAATCGATATACTCCGACTCCTCC	This study	4kb
Hel-ND4-F<br>Hel-CYTB-R	CAAAGTGATGGCAAATCATTGGCTGC<br>CTCTATAACTACCCCAAATTCACCATACC	This study	3.5kb
Hel-CYTB-F<br>Hel-12S-R	GGTATGGTGAATTTGGGGTAGTTATAGAG<br>TAGTAAGGAGAACTCTCCAAAACATGTTCC	This study	2.5kb
