# Supplementary material for: Monophyly of clade III nematodes is not supported by phylogenetic analysis of complete mitochondrial genome sequences
Source: BMC Genomics. 2011 Aug 3;12:392. doi: 10.1186/1471-2164-12-392 (PMC3163570; doi:10.1186/1471-2164-12-392)
Supplement: Additional File 5 — The species, taxonomy, and GenBank accession numbers for nematode species used in phylogenetic analyses in this study. [file 1471-2164-12-392-S5.PDF]

Additional file 5.
The species, taxonomic rank, and GenBank accession numbers for nematode species used in phylogenetic analyses in this study

Name of the species	Classification	GenBank accession number
Agamermis sp. BH-2006	Mermithida, Enoplea	NC_008231
Ancylostoma caninum	Rhabditida (Ancylostomatoidea), Chromadorea	NC_012309
Ancylostoma duodenale	Rhabditida (Ancylostomatoidea), Chromadorea	NC_003415
Anisakis simplex	Ascaridida (Ascaridoidea), Chromadorea	NC_007934
Ascaris sum	Ascaridida (Ascaridoidea), Chromadorea	NC_001327
Brugia malayi	Spirurida (Filarioidea), Chromadorea	NC_004298
Caenorhabditis briggsae	Rhabditida (Rhabditoidea), Chromadorea	NC_009885
Caenorhabditis elegans	Rhabditida (Rhabditoidea), Chromadorea	NC_001328
Chabertia ovina	Rhabditida (Strongyloidea), Chromadorea	NC_013831
Cooperia oncophora	Rhabditida (Trichostrongyloidea), Chromadorea	NC_004806
Cucullanus robustus¶	Ascaridida (Seuratoidea), Chromadorea	GQ332426
Dirofilaria immitis	Spirurida (Filarioidea), Chromadorea	NC_005305
Enterobius vermicularis	Oxyurida (Oxyuroidea), Chromadorea	NC_011300
Haemonchus contortus	Rhabditida (Trichostrongyloidea), Chromadorea	NC_010383
Heliconema longisimum¶	Spirurida (Physalopteroidea), Chromadorea	GQ332423
Heterorhabditis bacteriophora	Rhabditida (Rhabditoidea), Chromadorea	NC_008534
Hexamermis agrotis	Mermithida, Enoplea	NC_008828
Necator americanus	Rhabditida (Ancylostomatoidea), Chromadorea	NC_003416
Mecistocirrus digitatus	Rhabditida (Trichostrongyloidea), Chromadorea	NC_013848
Metastrongylus pudendotectus	Rhabditida (Metastrongyloidea), Chromadorea	NC_013813
Oesophagostomum dentatum	Rhabditida (Strongyloidea), Chromadorea	NC_013817
Onchocerca volvulus	Spirurida (Filarioidea), Chromadorea	NC_001861
Romanomermis culicivorax	Mermithida, Enoplea	NC_008640
Setaria digitata	Spirurida (Filarioidea), Chromadorea	NC_014282
Steinernema carpocapsae	Rhabditida (Strongyloidoidea), Chromadorea	NC_005941
Strelkovimermis spiculatus	Mermithida, Enoplea	NC_008047
Strongylus vulgaris	Rhabditida (Strongyloidea), Chromadorea	NC_013818

Strongyloides stercoralis	Rhabditida (Strongyloidoidea), Chromadorea	NC_005143
Syngamus trachea	Rhabditida (Strongyloidea), Chromadorea	NC_013821
Teladorsagia circumcincta	Rhabditida (Trichostrongyloidea), Chromadorea	NC_013827
Thaumamermis cosgrovei	Mermithida, Enoplea	NC_008046
Toxocara malaysiensis	Ascaridida (Ascaridoidea), Chromadorea	NC_010527
Trichinella spiralis	Trichocephalida, Enoplea	NC_002681
Trichostrongylus axei	Rhabditida (Trichostrongyloidea), Chromadorea	NC_013824
Wellcomia siamensis[¶]	Oxyurida (Oxyuroidea), Chromadorea	GQ332427
Xiphinema americanum	Dorylaimida, Enoplea	NC_005928
Lithobius forficatus	Myriapoda, Arthropoda	NC_002629
Limulus polyphemus	Chelicerata, Arthropoda	NC_003057

[¶]Species for those the complete mtDNA sequences are newly determined in this study.
